# Advances and challenges in personalized cardiac modeling and simulation for predicting the risk of sudden death from malignant arrhythmias in post-myocardial infarction

**DOI:** 10.3389/fcvm.2026.1879657

**Published:** 2026-10-15

**Authors:** Erwei Zhao, Liting Cheng, Huanfu Liu, Zefeng Wang, Yongquan Wu

**Affiliations:** Department of Cardiology, Capital Medical University Affiliated Anzhen Hospital, Beijing, China

**Keywords:** personalized heart modeling, risk stratification, sudden cardiac death, ventricular tachycardia, virtual electrophysiological study

## Abstract

Sudden cardiac death (SCD) represents a major global public health challenge, with malignant ventricular arrhythmias being its primary cause. This review focuses specifically on post-myocardial infarction (post-MI) ischemic cardiomyopathy—the most prevalent substrate for malignant ventricular arrhythmias. Current clinical practice predominantly relies on single functional indices, such as left ventricular ejection fraction, for risk stratification. This approach suffers from significant limitations in predictive accuracy, leading to dilemmas in the prophylactic application of implantable cardioverter-defibrillators, including both under-implantation and over-implantation. Personalized cardiac modeling and simulation technology, an advanced embodiment of the digital twin concept in cardiovascular medicine, offers a highly promising non-invasive solution to overcome these limitations. The core of this technology lies in the deep integration of personalized, multi-modal clinical imaging data, particularly late gadolinium enhancement sequences from cardiac magnetic resonance imaging. This enables the precise construction of individualized cardiac anatomical substrates that reflect the spatial distribution of myocardial fibrosis and scar. Building upon this anatomical foundation, multi-scale computational models are established, spanning from microscopic ion channel kinetics and cellular electrophysiological activity to macroscopic tissue conduction properties. These models systematically simulate core pro-arrhythmic factors under pathological conditions, including key ion channel remodeling, the coupling effects of non-myocytes, and alterations in anisotropic conduction properties of myocardial tissue. Ultimately, by implementing programmed electrical stimulation protocols on the computational heart model, the susceptibility to arrhythmias is dynamically assessed, achieving quantitative and visual stratification of an individual's SCD risk. This technological framework not only non-invasively reveals the dynamic electrical instability underlying static anatomical substrates but also demonstrates broad application prospects in understanding complex arrhythmia mechanisms, assisting in clinical therapeutic decision-making, and advancing the shift towards precision medicine models.

## 1 Background

Sudden cardiac death (SCD) is defined as an unexpected death from a cardiac cause, generally occurring within one hour of the onset of symptoms (1). While the global incidence ranges from 4 to 5 million cases per year, accounting for more than half of all cardiovascular deaths, even developed nations in Europe and North America witness about 350,000 SCD fatalities annually (2). According to estimates, the annual incidence of Sudden Cardiac Death (SCD) in China is approximately 540,000 cases, making it one of the most significant health problems in our society. Although predictors for SCD exist from multiple angles, there is no unified and accurate standard. In clinical practice, risk assessment based solely on Left Ventricular Ejection Fraction (LVEF) lacks precision, thereby resulting in both insufficient and excessive prophylactic implantation of Implantable Cardioverter-Defibrillators (ICDs). This scenario indicates that precise risk stratification to guide monitoring, resource allocation, and therapeutic decision-making remains a major unmet clinical need for patients affected by this complex and heterogeneous condition (3).

In recent years, digital twin technology has emerged as a transformative force in medicine. It utilizes computational models to simulate natural human structures, which are dynamically updated with data from their physical counterparts. This capability enables predictive analytics and informs high-value decision-making (4), aims to facilitate the early achievement of precision medicine by delivering management strategies tailored to individual patients (5). Cardiac simulation modeling, as an integral component of digital twin technology, plays a crucial role in the early prevention and management of sudden cardiac death (SCD). This approach has already yielded significant outcomes across various medical disciplines. For instance, in oncology, it utilizes multi-modal imaging and personalized radiobiological modeling to predict radiotherapy response in head and neck cancer (HNC) (6). In gynecology, it aids in the investigation and classification of conditions such as pre-eclampsia (7). Within cardiology itself, it assists in the prediction and diagnosis of cardiac conditions, including the study of atrial fibrosis substrates linked to atrial fibrillation (8) and forecasting the cardiovascular protective effects of novel lipid-lowering agents (9). Regarding the precise prediction of SCD, the critical leap from a “generic model” to a “personalized model” hinges on the assimilation and integration of multi-modal data. This process originates with high-resolution medical imaging, particularly Cardiac Magnetic Resonance (CMR) with Late Gadolinium Enhancement (LGE) sequences. These imaging data provide the personalized anatomical foundation for the model, encompassing cardiac geometry, wall thickness, and other structural details. Subsequently, employing well-established techniques from computational fluid dynamics and finite element analysis—such as image segmentation, 3D reconstruction, and mesh generation—these anatomical details are transformed into a geometric model suitable for numerical computation. The incorporation of electrophysiological properties relies on mapping cellular-level models onto the anatomical mesh. These properties are adjusted based on imaging characteristics to simulate pathological features like slowed conduction and increased dispersion of refractoriness in specific tissue regions. Finally, by applying simulated electrical stimuli to the heart model, the susceptibility to arrhythmias can be assessed non-invasively. This comprehensive technical framework marks the advent of a new era in cardiac research, transitioning into a phase of digital experimentation grounded in physics-based modeling.

## 2 Aims and distinct contribution of this review

While recent comprehensive reviews have summarized multiscale cardiac electrophysiology modeling and its clinical translation in sudden cardiac death risk prediction (e.g., Trayanova et al., Physiol Rev 2024), the present review focuses specifically on personalized post–myocardial infarction ventricular modeling for SCD risk stratification in ischemic cardiomyopathy. Unlike previous overviews that broadly survey models across multiple disease entities, this article's main contribution is to provide a systematic introduction to, and side-by-side comparison of, alternative strategies for key steps in the post-MI modeling pipeline—including anatomical reconstruction, ionic remodeling parameterization, tissue-level fibrosis representation, and conduction velocity personalization—together with a discussion of the applicability and limitations of each approach and incorporation of the latest methodological advances. By consolidating these methodological choices into a coherent, critically appraised workflow, this review serves as a practical, method-oriented guide for implementing image-based ventricular simulations to support personalized SCD risk assessment in patients with ischemic cardiomyopathy.

## 3 SCD

### 3.1 The current situation of SCD

#### 3.1.1 The mechanism of SCD

The underlying mechanism of sudden cardiac death (SCD) is primarily ventricular arrhythmias, with ventricular fibrillation and ventricular tachycardia accounting for the vast majority of cases. These malignant ventricular arrhythmias occur in the setting of various triggers and substrates. The most common substrate is myocardial scar, which can result from either ischemic injury or various non-ischemic insults (10). In individuals over 35 years of age, coronary artery disease is the predominant cardiac condition leading to these malignant arrhythmias and represents the most frequent cause of SCD, responsible for over 80% of cases.

As the most common condition leading to SCD, coronary artery disease (CAD) contributes to SCD risk through multiple distinct mechanisms, which can be categorized into those occurring during the acute infarction phase and those manifesting post-infarction. During the infarction phase, often triggered by plaque rupture, acute myocardial ischemic injury occurs. This setting is predominantly associated with primary ventricular fibrillation (VF), although other primary arrhythmias such as Torsades de Pointes (TdP), bradyarrhythmias, and monomorphic ventricular tachycardia (VT) may also be observed. The second major mechanism involves post-infarction scar-related ventricular tachycardia. Following myocardial infarction (MI), necrotic cardiomyocytes trigger an acute inflammatory response characterized by the recruitment of inflammatory cells and the release of pro-inflammatory cytokines. As inflammation gradually resolves, transforming growth factor-β (TGF-β) signaling becomes activated, promoting fibroblast activation and differentiation into myofibroblasts. These myofibroblasts synthesize and deposit large amounts of collagen and other extracellular matrix (ECM) components, leading to fibrotic scar formation that replaces the lost myocardium and preserves the structural integrity of the ventricular wall (11). The formation of a myocardial scar following an infarction provides a critical structural substrate for re-entry circuits by creating areas of unidirectional block and slowed conduction. This pathophysiological basis is corroborated by cardiac magnetic resonance (CMR) studies, where scar heterogeneity stands as a strong predictor for ventricular arrhythmias (12). A conceptual framework summarizing the mechanistic transition from myocardial infarction-induced remodeling to ventricular arrhythmogenesis and SCD is illustrated in Figure 1.

**Figure 1:**
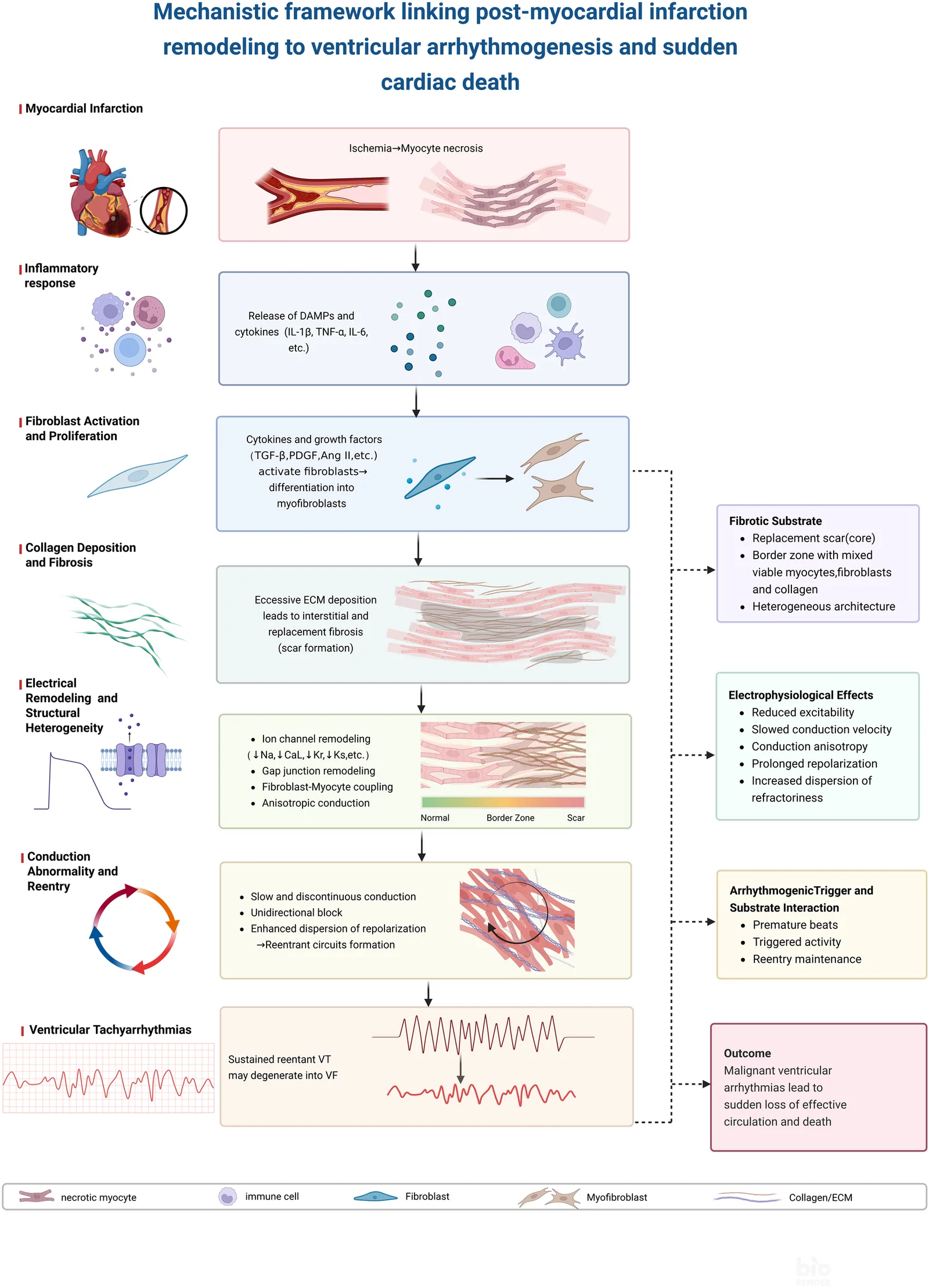
Mechanistic framework linking post-myocardial infarction remodeling to ventricular arrhythmogenesis and sudden cardiac death: myocardial infarction initiates inflammatory activation, fibroblast proliferation, and extracellular matrix deposition, resulting in scar formation and heterogeneous border-zone remodeling. Structural and electrical remodeling alter conduction properties and repolarization dynamics, promoting conduction slowing, unidirectional block, and reentrant ventricular arrhythmias. Created in BioRender. ZHAO, E. (2026) https://BioRender.com/uzud7yi.

Myocardial scar is a manifestation of ventricular fibrosis. Fibrosis involves fibroblast activation and ECM collagen deposition; when excessive, it becomes maladaptive (13). Fibrosis manifests in two primary forms: replacement and reactive interstitial fibrosis. Replacement fibrosis is a response to cardiac injury (e.g., post-myocardial infarction), activating a repair reaction where dead cardiomyocytes are replaced by a collagen-based scar. In contrast, reactive interstitial fibrosis is characterized by an expansion of the cardiac interstitial space without substantial cardiomyocyte loss. In the post-MI context, the border zone surrounding the replacement scar—where surviving myocytes intermingle with collagen deposits—creates a particularly arrhythmogenic substrate, as it harbors critical components for re-entrant circuits and fosters a milieu favorable for the initiation of arrhythmic triggers. Under disease states, adverse remodeling and myocardial fibrosis can become a self-perpetuating process, leading to progressive distortion of cardiac structure and function. This results in increased mechanical stiffness, diastolic dysfunction, and creates a substrate that supports arrhythmogenesis (14).

Fibrotic patterns are further categorized by their texture: compact, patchy, interstitial, and diffuse (15). Compact fibrosis, characterized by acellular areas with dense collagen deposition, possesses the least arrhythmogenic potential among all types, as the scar itself does not inherently increase susceptibility to unidirectional block or slow conduction. However, when other substrate conditions are present, it can provide an area of fixed block to anchor re-entrant circuits (16).

Interstitial and patchy fibrosis exhibit the highest arrhythmogenic potential. These patterns involve regions where bundles of viable myocytes are separated by collagenous septa, with tenuous connections between surviving cells. This microstructure can cause both significant regional conduction slowing (17, 18) and unidirectional block due to source-sink mismatch (19). Diffuse fibrosis features short collagen strands interspersed among myocardial fibers. These strands increase the anisotropy of conduction by selectively disrupting side-to-side gap junction coupling between cardiomyocytes, thereby also elevating arrhythmic risk (16). Among these patterns, patchy and interstitial fibrosis are most relevant to the post-infarction border zone, where the intermingling of viable myocyte bundles and collagen septa creates the conditions for source-sink mismatch and unidirectional block that underlie reentrant ventricular tachycardia.

While SCD encompasses a diverse spectrum of etiologies—including inherited channelopathies, hypertrophic cardiomyopathy, dilated cardiomyopathy, and coronary artery disease—the most common underlying substrate in adults over 35 years of age is ischemic heart disease, accounting for over 80% of SCD cases. Among ischemic etiologies, post-myocardial infarction (post-MI) scar-related ventricular arrhythmias represent the most extensively studied and clinically actionable substrate for personalized computational modeling. The structural and electrical remodeling following MI—namely, the infarct scar and its heterogeneous border zone—creates a well-defined arrhythmogenic substrate that is uniquely amenable to image-based reconstruction and simulation. Accordingly, this review focuses specifically on post-MI ischemic cardiomyopathy, while acknowledging that many of the modeling methodologies discussed may be adapted to other disease contexts.

#### 3.1.2 Clinical detection methods

In clinical practice, the assessment of sudden cardiac death (SCD) risk and the stratification of its severity can be broadly classified into invasive and non-invasive approaches.

##### 3.1.2.1 Non-invasive approaches

Non-invasive approaches include techniques such as the 12-lead electrocardiogram (ECG), exercise stress testing, Holter monitoring, cardiac imaging (including echocardiography and cardiac magnetic resonance, among others), and genetic testing.

1. Resting 12-lead ECG

The resting 12-lead electrocardiogram (ECG) is a useful tool for the diagnosis and screening of patients at increased risk of sudden cardiac death (SCD). As many underlying conditions involve disturbances in the heart's electrical conduction, this diagnostic tool can provide significant insight into the underlying pathology and its progression, offering etiological clues in patients with potential SCD risk. Fragmented QRS (fQRS) on 12-lead ECG reflects inhomogeneous ventricular activation and predicts myocardial scar and mortality in both ischemic and non-ischemic cardiomyopathy (20). In post-infarction patients, fQRS has been specifically associated with the presence of intramural scar and the extent of border zone tissue, making it a readily accessible marker of the arrhythmogenic substrate. However, ECG findings are often non-specific for SCD itself, and further supporting evidence is required to accurately diagnose the risk of this condition (21).

2. Ambulatory ECG

Holter monitoring detects arrhythmic events and reflects autonomic tone via indices such as Heart Rate Variability (HRV), Heart Rate Turbulence (HRT), and Baroreflex Sensitivity (BRS). Repolarization heterogeneity, quantified as microvolt T-wave alternans (TWA), is also linked to SCD risk (22). Evidence indicates that reduced HRV is associated with a higher incidence of ventricular arrhythmias and increased mortality. Beyond HRV, several large-scale retrospective and prospective studies have provided strong evidence that HRT is a powerful independent predictor of post-AMI risk (23). While these dynamic ECG parameters hold value in risk prediction, and Holter monitoring is recommended for patients with relevant symptoms, its limitations are similar to those of a standard resting ECG: it cannot specifically characterize the patient's underlying cardiac structural substrate, and abnormal findings typically necessitate further investigation for a comprehensive risk assessment. Furthermore, in post-infarction patients, Holter monitoring cannot distinguish whether detected non-sustained ventricular tachycardia originates from the scar border zone or from the remote myocardium, limiting its ability to localize the arrhythmogenic substrate.

3. Echocardiography and Cardiac Magnetic Resonance

Echocardiography and cardiac magnetic resonance (CMR) provide precise diagnostic capabilities for structural heart disease. Abnormalities in left ventricular geometry are associated with an increased risk of sudden cardiac death (SCD), even in patients with preserved left ventricular ejection fraction. This represents one of the most valuable predictive aspects offered by echocardiography. In post-infarction patients, echocardiography is routinely used to assess left ventricular ejection fraction (LVEF), wall motion abnormalities, and global remodeling. However, despite the predictive value of identifiable structural abnormalities for SCD, the population attributable risk (PAR) for most SCD events lies within subjects without any known structural heart disease，but with specific arrhythmogenic substrate related to myocardial infarction This fact limits the efficiency of relying solely on structural imaging for general SCD screening in the broader population (24).

Cardiac magnetic resonance (CMR) with late gadolinium enhancement (LGE) enables the characterization of myocardial fibrosis and scar tissue. Myocardial fibrosis is a determinant of the arrhythmogenic predisposition in cardiomyopathies and has been demonstrated to be a stronger predictor of ventricular arrhythmias than the degree of left ventricular (LV) systolic dysfunction. The presence of LGE is considered to reflect the accumulation of collagen in the extracellular space, thereby serving as an imaging surrogate for fibrosis. It is noteworthy, however, that LGE can also be present in settings without a fibrotic substrate, including myocardial edema or infiltrative diseases (22). In post-infarction patients, LGE-CMR is particularly valuable because it can delineate the infarct core, the peri-infarct border zone, and the remote myocardium—three tissue compartments with distinct electrophysiological properties that collectively determine arrhythmic risk. Beyond the general assessment of LGE, other MRI characteristics of myocardial scar, such as scar heterogeneity (quantified using parameters like the border zone, gray zone, or scar entropy) or specific locational features (e.g., a ring-like fibrosis pattern), have also been linked to an increased risk of SCD (22). However, while CMR can visualize certain complexities of arrhythmogenic myocardial scar, it cannot independently assess the physiology essential for initiating and sustaining ventricular arrhythmias. Critical characteristics of channels within the scar, such as abnormal conduction velocity, abnormal refractory periods, and decremental conduction behavior—which are crucial for mediating re-entry—are not visible on imaging (10). Moreover, there is currently no unified consensus regarding the impact of the extent, location, and pattern of LGE on clinical outcomes (25). Finally, a standardized methodology for myocardial scar quantification has yet to be established. Most predictive CMR techniques for SCD risk stratification are based on a qualitative analysis, where areas of infarction or fibrosis (scar) are identified by a signal intensity (SI) higher than that of normal myocardium after gadolinium administration (Late Gadolinium Enhancement, LGE) (24).

The presence of Late Gadolinium Enhancement (LGE) has been demonstrated to be closely associated with the risk of malignant ventricular tachyarrhythmias and sudden cardiac death (SCD), with its extent, location, and heterogeneity serving as critical risk markers. In post-infarction patients, the spatial relationship between the LGE-positive scar and the adjacent viable myocardium is particularly important, as reentrant circuits typically involve conduction channels within the border zone that connect regions of healthy tissue. Heterogeneous LGE likely reflects an admixture of viable cardiomyocytes and scar tissue, which can create conditions conducive to the formation of bypass tracts or lead to regional electrophysiological abnormalities. Conversely, extensive LGE may disrupt electrical conduction, thereby inducing ventricular tachyarrhythmias and contractile dysfunction.

4. Genetic test

Genetic testing is commonly employed for predicting cardiomyopathies and inherited ion channelopathies. In these conditions, the heart is structurally normal, with the pathology residing at the molecular level due to mutations in genes encoding cardiac ion channel proteins. These channelopathies increase the risk of ventricular fibrillation due to inherent electrical instability. Therefore, clinical screening and genetic testing of family members are crucial in cases of non-structural SCD (1). Risk stratification can be performed based on the specific gene mutated, the location of the mutation, and its functional type. Concurrently, research has indicated a genetic component to Coronary Artery Disease (CAD), with its heritable predisposition measurable by a dedicated polygenic risk score (PRS). However, because the associated risk is non-linear, a meaningfully elevated risk is observed only in a small subset of the patient population (26).

##### 3.1.2.2 Invasive approach

Invasive approaches primarily refer to Electrophysiological Study (EPS), a procedure that involves the use of catheters to induce ventricular arrhythmias within the cardiac chambers and record subsequent electrical activity. Its main application is to guide catheter ablation procedures or to diagnose ventricular arrhythmias of uncertain etiology when other methods have failed; it is rarely used solely for prediction. The primary value of EPS lies in risk stratification and prognosis assessment, particularly for patients with structural heart disease, such as those post-myocardial infarction. The inducibility of sustained monomorphic ventricular tachycardia (SMVT) during EPS is associated with an increased risk of subsequent spontaneous ventricular arrhythmic events. Importantly, early landmark trials established the clinical relevance of EPS-guided risk stratification in patients with coronary artery disease. In the Multicenter Automatic Defibrillator Implantation Trial (MADIT-I), patients with prior myocardial infarction, left ventricular dysfunction, asymptomatic nonsustained ventricular tachycardia, and inducible, nonsuppressible ventricular tachyarrhythmia during EPS experienced improved overall survival with prophylactic ICD implantation compared with conventional medical therapy (27). Similarly, the Multicenter UnSustained Tachycardia Trial (MUSTT) demonstrated that EPS could identify patients with coronary artery disease, reduced left ventricular function, and nonsustained ventricular tachycardia who were at increased risk of arrhythmic death or cardiac arrest, and that outcomes were influenced by subsequent electrophysiologically guided therapy, particularly ICD therapy (28). However, a major limitation is that non-inducibility for ventricular fibrillation (VF) does not equate to a low-risk status, as these patients may still develop VF due to acute triggers like ischemia or heart failure. Consequently, although EPS provides a direct recording of cardiac electrical activity, its reliability for predicting ventricular arrhythmias is not absolute. A *post-hoc* analysis of the Multicenter Automatic Defibrillator Implantation Trial II (MADIT II) indicated that EPS had limited predictive value for future ventricular tachycardia or fibrillation events (29). Therefore, while EPS cannot be used as a standalone predictive method for SCD, its results hold significant value in guiding subsequent therapies, notably radiofrequency catheter ablation.

Given the multitude of risk factors contributing to sudden cardiac death (SCD)—including alterations in the ventricular substrate, autonomic nervous dysfunction, and ion channel abnormalities—the effective identification and integration of such data are crucial for the individualized prediction of SCD events and the subsequent selection of preventive strategies. The current primary criterion guiding clinical prevention strategies is the Left Ventricular Ejection Fraction (LVEF). While LVEF provides a direct and effective measure of systolic function, its utility for predicting ventricular arrhythmias resulting from multifactorial causes is limited, and it may not be fully applicable to every individual. This can lead to either under-prevention or over-prevention (30). For instance, many women who experience SCD lack traditional risk factors, such as a severely reduced LVEF or a established diagnosis of coronary artery disease (CAD) prior to the event (31). Concurrently, a significant number of patients with only mild to moderate reductions in LVEF may still be susceptible to SCD (32), presenting a substantial challenge to contemporary SCD prediction paradigms.

In summary, despite the existence of various SCD risk stratification tools, their predictive efficacy is limited by the singularity of assessment dimensions and the fragmentation of information types. Functional metrics represented by left ventricular ejection fraction fail to reveal structural substrates; structural imaging exemplified by LGE-CMR struggles to translate into dynamic electrical instability; and while invasive electrophysiological studies can directly assess electrophysiological properties, their invasive nature and sampling limitations hinder their use in widespread screening. This “function-structure-electrophysiology” assessment gap constitutes a major obstacle to the precise prevention of SCD. Consequently, there is an urgent need in the field to develop a new method that is non-invasive, integrates multimodal data, and quantitatively assesses cardiac electrophysiological stability. Personalized cardiac simulation modeling has emerged precisely in response to this demand as a transformative technological paradigm.

Table 1 presents a comparison between different methods.

**Table 1:** Comparison between different detection methods.

Category	Traditional non-invasive method	Traditional invasive methods	Simulation modeling
Assessment dimension	Static structure & function	Local electrophysiological response	Dynamic, integrated electrophysiological substrate
Primary output	Descriptive diagnostic data	Invasive diagnostic conclusion	Mechanism-based decision support
Advantages	Non-invasive & safe, easily repeatable. Technically mature, routine clinical use. Guideline-endorsed, widely available.	One of the gold standards in electrophysiology. Can directly induce clinical arrhythmias. Enables real-time guidance for ablation therapy.	Non-invasive, enabling a “comprehensive virtual EPS”. Highly personalized with mechanistic insight. Provides prospective guidance for therapy optimization and pre-procedural planning.
Limitations	Information “silos”, difficult to integrate. Cannot assess electrophysiological substrate stability. Predictive ceiling for SCD exists.	Invasive procedure with associated risks. Sampling limitation, potential for missed diagnosis. Cannot predict risk from non-induced zones. Difficult to repeat, not suitable for screening.	Not yet routine, undergoing clinical validation. Model complexity requires expertise and computational resources. Lacks a unified validation gold standard.
Exemplary technologies	LGE-CMR, echocardiography. 12-lead ECG, embulatory ECG (Holter).	3D electroanatomic mapping.	Personalized 3D cardiac geometric reconstruction.

## 4 Cardiac modeling and simulation

Cardiac computational modeling has evolved from models of individual cardiac cells to increasingly integrated representations of cardiac structure and electrophysiology. With advances in imaging, computational methods, and physiological modeling, cardiac simulations can now incorporate information across multiple spatial scales to investigate the mechanisms underlying cardiac electrical activity and arrhythmogenesis. In the context of post-MI ventricular modeling, this multiscale framework provides a basis for integrating structural and electrophysiological characteristics into a personalized representation of the diseased heart (Figure 2). The development of these approaches has been accompanied by continuous advances in both modeling theory and computational techniques, which have progressively expanded their applications from basic physiological investigation to patient-specific arrhythmia research.

**Figure 2:**
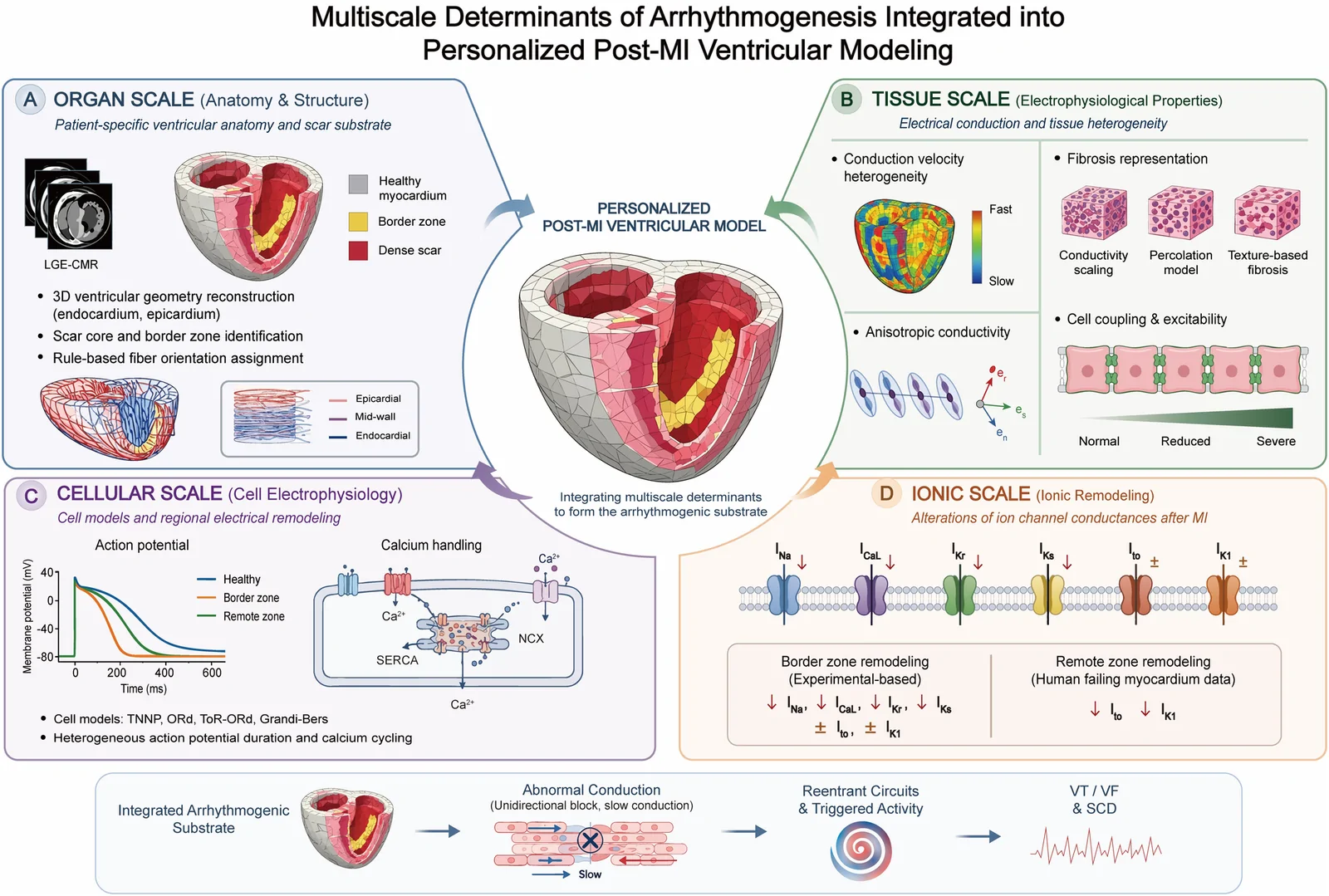
Multiscale determinants of arrhythmogenesis integrated into personalized post-MI ventricular modeling: multiscale determinants of arrhythmogenesis integrated into personalized post-myocardial infarction (post-MI) ventricular modeling. **(A)** Organ scale: patient-specific ventricular anatomy, infarct scar and border-zone geometry, together with myocardial fiber orientation, are reconstructed from imaging data, particularly late gadolinium enhancement cardiac magnetic resonance (LGE-CMR). **(B)** Tissue scale: regional electrophysiological heterogeneity is represented through spatial variations in conduction velocity, anisotropic conductivity, and fibrosis-related tissue properties, including different computational representations of fibrotic tissue. **(C)** Cellular scale: cellular electrophysiological models capture regional differences in action potential characteristics and calcium handling, with heterogeneous cellular excitability across healthy, border-zone, and remote myocardial regions. **(D)** Ionic scale: post-MI ionic remodeling is represented by changes in major transmembrane ion-channel and calcium-handling properties, including sodium, calcium, and potassium currents and sarcoplasmic reticulum calcium cycling. Integration of these multiscale structural and electrophysiological determinants produces a personalized ventricular model that captures the heterogeneous arrhythmogenic substrate. The resulting substrate may promote abnormal conduction, unidirectional block, reentrant circuits, and triggered activity, ultimately contributing to ventricular tachycardia/ventricular fibrillation (VT/VF) and sudden cardiac death (SCD).

### 4.1 History

Cardiac computer simulation modeling emerged relatively early abroad. The University of Oxford pioneered the first myocardial cell model, established the theoretical framework for multi-scale cardiac physiological and pathological research, and collaborated with the University of Auckland to develop simulation software that incorporated mechanical properties into cardiac modeling. Furthermore, institutions such as Utrecht University conducted foundational research into the mechanisms of arrhythmias, laying the theoretical groundwork for cardiac simulation. At the microscopic scale, Johns Hopkins University integrated data on calcium homeostasis, cell signaling, and energy metabolism to develop cell-level electro-mechano-metabolic models for investigating the ionic mechanisms underlying arrhythmogenesis. At the macroscopic scale, their research involves constructing multi-scale heart models by incorporating personalized disease states and clinical images to extract myocardial fiber orientation (33). Domestically, Zhejiang University created the world's first whole-heart model incorporating a complete conduction system. This model has been utilized to study normal electrical excitation propagation, atrial fibrillation, ECG genesis, and body surface potential maps. Additionally, in collaboration with Beijing Anzhen Hospital, Capital Medical University, they are conducting research to explore the clinical feasibility of this technology.

### 4.2 Theory

The genesis of the cardiac action potential (AP), representing the electrical activity of a single cardiomyocyte, requires the coordinated activation of a sophisticated array of ion channels, pumps, and exchangers to produce a well-defined electrical impulse (34). At the single-cell level, the electrical AP triggers mechanical contraction by inducing a transient rise in intracellular calcium concentration, which subsequently activates the cell's contractile proteins. This process of coupling electrical excitation to mechanical action is termed excitation-contraction coupling. APs generated within individual cells propagate from cell to cell via gap junctions, forming waves of excitation that activate and synchronize the heart's pumping function (35). The resting potential of ventricular myocytes is primarily maintained by potassium efflux through potassium channels, with the potassium concentration gradient dependent on the continuous activity of the Na^+^/K^+^-ATPase pump, stabilizing the membrane potential at approximately −90 mV. During Phase 0(rapid depolarization) of the AP, voltage-gated sodium channels activate in response to mild depolarization from an external stimulus or electrotonic interaction from a neighboring cell. This results in a massive influx of sodium ions, generating the fast sodium current (I_Na) that drives rapid depolarization and subsequently activates other voltage-dependent ion channels (36). The most rapidly activating current is the transient outward potassium current (I_to), which causes a brief, rapid efflux of potassium ions before quickly inactivating, contributing to the early rapid repolarization of Phase 1. Phase 2, the plateau phase, is critically sustained by the balance between the influx of calcium ions through L-type calcium channels and the efflux of potassium ions through delayed rectifier potassium channels (37). Simultaneously, the Na^+^/Ca^2+^ exchanger contributes to calcium homeostasis. During Phase 3(rapid repolarization), L-type calcium channels inactivate, while delayed rectifier potassium channels remain open, allowing potassium efflux to dominate and restore the negative membrane potential. In Phase 4(resting phase), the inward rectifier potassium current (I_K1) is the primary conductance responsible for maintaining the stable resting membrane potential (37). It is important to note that the APs in ventricular working cardiomyocytes are not automatic; they are electrically triggered by impulses originating from the pacemaker cells of the sinoatrial node via the cardiac conduction system.

Cellular depolarization triggers mechanical contraction primarily through excitation-contraction coupling. During the AP plateau (Phase 2), the small amount of calcium entering the cell via L-type calcium channels binds to ryanodine receptors on the sarcoplasmic reticulum (SR). This binding triggers a massive release of calcium from the SR into the cytoplasm, a process known as calcium-induced calcium release (CICR). The released calcium ions bind to troponin C, initiating the cross-bridge cycling and sliding of actin and myosin filaments, culminating in myocardial contraction (38).

Following contraction and repolarization, the cardiomyocyte must enter a refractory period during which it is unable to depolarize again. This period is defined as the interval after excitation during which the cell remains inexcitable or requires a supranormal stimulus to be re-excited. This state is primarily due to the temporary inactivation of sodium channels (responsible for the depolarizing current in working myocardium). The refractory period is classified as absolute (completely unexcitable) or relative (excitable only by a stronger-than-normal stimulus) (39).

Myocardial tissue models are an extension of single-cell models, formed by connecting surrounding cells via gap junctions. These junctions, which are specialized membrane structures composed of intercellular ion channels, play a critical role in the successful propagation of electrical impulses by facilitating both electrical and chemical communication between cells (39). This enables changes in membrane potential within a single cell to be transmitted to adjacent cells through these junctions, triggering subsequent action potentials and generating electrical currents at the tissue level. Furthermore, the myocardium is not a homogeneous tissue but a composite structure composed of multiple layers of aligned myocardial fibers. The ventricular wall extends from the endocardium to the epicardium, with the orientation of myocardial fibers changing in a regular pattern across these layers, creating a laminar architecture. This specific arrangement results in anisotropic conduction of electrical signals, where the conduction velocity along the longitudinal axis of the fibers is significantly faster than that along the transverse axis. At the cellular level, this anisotropy arises because gap junctions are predominantly concentrated at the end-to-end connections between cardiomyocytes, with fewer lateral connections, resulting in lower electrical resistance along the longitudinal direction compared to the transverse direction (40).

Common mechanisms of arrhythmogenesis include early afterdepolarizations (EADs), triggered activity, and reentry. While EADs and triggered activity can be simulated at the cellular level, reentry is most commonly observed in larger myocardial tissues. Under normal conditions, myocardial electrical activation propagates from its source in an orderly, unidirectional manner until the entire tissue is activated. The time delay between activation and repolarization of the cardiac action potential creates a region of refractory tissue behind the wavefront, which normally prevents re-excitation. Reentry becomes possible if a portion of the myocardium, initially bypassed by the wavefront due to an altered substrate, subsequently recovers its excitability. This allows the impulse to re-enter and propagate through this region, establishing a self-sustaining reentrant circuit. For reentry to persist, each segment of the circuit must recover excitability before the arrival of the next wavefront. Therefore, the maintenance of a reentrant circuit depends on a critical balance between conduction velocity (CV) and the refractory period, a condition more likely to occur in electrically and structurally heterogeneous tissues (41). This highlights the clinical importance of timely identification of altered ventricular substrates. Since most ventricular arrhythmias are initiated by reentry, a key focus of model development is the accurate representation of the specific electrophysiological conditions that facilitate this mechanism.

The clinical gold standard for assessing the myocardial substrate is late gadolinium enhancement (LGE) cardiac magnetic resonance imaging. Quantitatively, fibrotic tissue is commonly identified by applying a signal intensity threshold—for example, pixels exceeding 5 standard deviations above the mean signal intensity of reference normal myocardium are classified as scar (42). This threshold-based classification directly informs the anatomical reconstruction step described in Section 4.3.1.

### 4.3 Method

#### 4.3.1 Anatomical reconstruction

In cardiac simulation modeling, anatomical reconstruction is the foundational step. Magnetic resonance imaging (MRI), due to its high sensitivity for soft tissues, is often the first-line modality for cardiac anatomical reconstruction. The key task is to convert these images into a computational mesh suitable for simulation. Two principal segmentation approaches are used: semi-automatic segmentation and fully automatic segmentation.

The resolution of current clinical MRI is relatively low, with in-plane resolution approximately 1.5 mm and through-plane resolution around 10 mm. A typical cardiac scan yields about 10 image slices, making automatic segmentation of the ventricular walls challenging. Consequently, semi-automatic segmentation of the ventricles is commonly employed for anatomical reconstruction. The process typically begins with manually selecting feature points on the epicardial and endocardial borders in the clinical CMR images. These points are then used to automatically fit the inner and outer surfaces of the ventricles. The second step involves applying a variational implicit function method to automatically interpolate the segmented cardiac cavity surfaces, thereby generating high-resolution volumetric data of the cavities. Following this, a Gaussian mixture model is utilized to automatically segment the infarct region. The segmented tissues are classified into core infarct and border zone (or partial infarction) areas. A probabilistic logarithmic algorithm is then employed to interpolate the segmented infarct regions, producing high-resolution data of the infarcted tissue. Finally, the high-resolution image data undergoes finite element mesh generation. Using a rule-based automatic construction algorithm, the myofiber orientation, including the helical fiber arrangement within the patient's myocardium, is established. This comprehensive process yields a personalized, anatomically reconstructed cardiac model (43). Despite its widespread use, semi-automatic segmentation is labor-intensive and introduces inter-observer variability, as the manual selection of feature points depends on operator expertise. This limits its applicability to large-cohort studies and may introduce bias in multicenter comparisons.

Recent studies have demonstrated the feasibility of fully automatic ventricular segmentation. Yet fully automatic segmentation of the ventricle and infarct scar from LGE−MRI does not rest on a single canonical pipeline, as threshold-based, atlas-based, and deep-learning-based tools differ substantially in how they localize the heart, delineate the myocardial wall, label enhanced pixels, subdivide remote myocardium from border zone and scar core, and finally project the result onto a simulation mesh. What these methods share is a conceptual continuum rather than a fixed recipe: cardiac localization is followed by myocardium boundary delineation, then by enhanced-pixel detection within the myocardial ROI, tissue classification into remote–border–core compartments, and finally geometric projection with optional conduction-channel extraction for electrophysiological use.

Semi-automatic segmentation offers greater operator control in ambiguous cases and has been widely validated in proof-of-concept studies. Fully automatic approaches improve reproducibility and throughput, making them preferable for population-scale studies when image quality is adequate. Hybrid workflows—initial auto-segmentation followed by minimal manual correction—are increasingly regarded as a pragmatic compromise for balancing accuracy and scalability in clinical translation.

#### 4.3.2 Setting electrophysiological parameters

After obtaining the geometric model, the electrical activity of the heart requires separate modeling and adjustment of electrophysiological parameters. There are several methods to choose from for both basic cardiac modeling and parameter adjustment.

Several human ventricular cell models have been developed for cardiac electrophysiology simulation, including the ten Tusscher–Noble–Panfilov (TNNP) model (44), the O'Hara–Rudy (ORd) model (45), and the Grandi–Bers model (46). These models were not formulated arbitrarily: the TNNP model was constructed from recent experimental data on human ventricular myocytes and heterologously expressed human ion channels, and its action potential morphology, APD90 (≈270 ms at 1 Hz, within the experimentally reported 270–350 ms range for human epicardial myocytes), APD restitution shape, and tissue conduction velocity restitution were used as validation targets. The ORd model was later validated against APD rate-dependence and restitution recorded from over 100 undiseased human hearts together with new human ventricular ICaL, IKr, IKs, INaCa and IK1 measurements. The Grandi-Bers model was validated against human atrial and ventricular AP characteristics compiled from multiple isolated-myocyte studies. Comparative analyses have shown that these models differ in AP morphology, APD restitution, and sensitivity to ionic perturbations, with the TNNP and appropriately configured ORd models exhibiting the closest agreement with experimental data under ischemic conditions (47, 48). More recently, the Tomek–Rodriguez variant of the O'Hara–Rudy model (ToR-ORd) refined the original ORd formulation by revisiting the L-type calcium current driving force, replacing the rapid delayed rectifier potassium current (I∼Kr∼) with a Markov model, updating I∼Na∼, I∼K1∼, and chloride currents based on human data, and improving agreement with experimentally measured action potential morphology and calcium handling (49).

Several human ventricular cellular electrophysiological models have been developed to represent cardiac action potential dynamics, with differences in their ionic formulations and physiological targets. Representative models discussed in this review are summarized in Table 2.

**Table 2:** Representative human ventricular cellular electrophysiological models used in cardiac computational modeling.

Model	Year	Main characteristics	Key electrophysiological features	Relevance to post-MI modeling
TNNP	2004	Human ventricular action potential model with detailed ionic formulation	Represents major ionic currents and ventricular AP dynamics	Widely used as a baseline framework for incorporating disease-related ionic remodeling
O'Hara–Rudy (ORd)	2011	Experimentally validated human ventricular myocyte model	Detailed representation of ionic currents, Ca^2+^ handling, and AP morphology	Provides a widely used framework for modeling regional electrophysiological heterogeneity
Grandi–Bers	2010	Human ventricular model with detailed excitation–contraction coupling	Detailed Ca^2+^ cycling and Na^+^/Ca^2+^ handling	Useful when post-MI remodeling involves altered Ca^2+^ handling and excitation–contraction coupling
ToR-ORd	2019	Calibrated human ventricular model developed from ORd	Improved representation of human ventricular electrophysiology across physiological and pathological conditions	Provides a more recently calibrated framework for disease-specific and drug-related modeling

##### 4.3.2.1 Cell model parameters and what measurements are available to validate their choice

Within the post-infarction context, the most extensively characterized cellular parameter set is that of the infarct border zone (BZ). Research indicates that the tissue surrounding a scar formed after an ischemic event undergoes remodeling during the healing process as it transitions into the chronic phase, leading to alterations in the properties of its ion channels. This remodeled tissue, termed the infarct border zone (BZ), creates a substrate conducive to reentrant circuits and abnormal propagation. These changes result in modifications to the action potential duration (APD) and calcium kinetics, which can promote abnormal action potential (AP) propagation and the genesis of reentry.

In the BZ, the most consistently observed ionic alterations include a marked reduction in the sodium current (I_Na) and the L-type calcium current (I_CaL), as well as moderate downregulation of the rapid and slow delayed rectifier potassium currents (I_Kr, I_Ks) (50). These changes collectively lead to a prolonged APD, a reduced maximum depolarization velocity, and a lower plateau potential in BZ myocytes compared to healthy tissue (51). To quantitatively determine the appropriate degree of conductance reduction for each ion channel, computational models have been employed to fit these experimentally observed changes.

To quantitatively determine the appropriate degree of conductance reduction for each ion channel, the most widely cited human-adapted representation of infarct border zone (BZ) ion channel remodeling was introduced by Arevalo et al. (2016), in which the ten Tusscher–Panfilov(TNNP) human ventricular myocyte model was used as the baseline, and ion conductances in the grey zone (GZ) were downregulated according to canine EBZ patch-clamp data—62% reduction in I_Na, 69% in I_CaL, 70% in I_Kr, and 80% in I_Ks—producing prolonged APD (360 vs. 310 ms), reduced V˙max, and lowered plateau potential. Limitations of this scheme are severalfold. First, the scaling factors are extrapolated from canine epicardial border zone patch-clamp recordings to a human ventricular ionic background, assuming that relative conductance reductions are conserved across species despite known quantitative differences in baseline current densities and the scarcity of direct human BZ patch-clamp data. Second, the grey zone is assigned a spatially homogeneous remodeling profile, neglecting the continuous transmural and circumferential ionic gradients that exist between the necrotic core and remotely surviving myocardium. Third, several remodeling features documented in human failing or post-MI myocardium are omitted, including late sodium current (I_NaL) upregulation, Na^+^/Ca^2+^ exchanger (NCX) enhancement, SERCA downregulation, and CaMKII-dependent modulation of calcium handling. Fourth, fibroblast–myocyte electrical coupling is not included, which may substantially affect conduction velocity and re-entry susceptibility in the peri-infarct region. Finally, acute ischaemic metabolic factors—such as extracellular hyperkalaemia, acidosis, and ATP-sensitive potassium current (I_K(ATP)) activation—are absent, even though these dominate the electrophysiological substrate during the earliest post-occlusion phase (52).

Although illustrated here with the TNNP (Ten Tusscher–Panfilov 2006) human ventricular myocyte model, the same canine-derived relative conductance reductions—expressed as multiplicative scaling factors—are routinely applied to other biophysically detailed human ventricular models (e.g., ToR-ORd, Grandi–Bers) by scaling each model's baseline maximal conductances, under the explicit acknowledgement that this constitutes a species extrapolation rather than direct human border-zone measurement.

Unlike canine epicardial border zone remodeling schemes derived from Decker and Rudy (2010) (53), Beuckelmann et al. (1993) directly measured K^+^ currents in isolated human ventricular myocytes from patients with terminal heart failure (ischemic and dilated cardiomyopathy) using whole-cell patch-clamp. They reported a ∼36% reduction in transient outward K^+^ current (I_to) density (9.1→5.8 *μ*A/cm^2^, *P* < 0.01) and a ∼47% reduction in inward rectifier K^+^ current (I_K1) density (−15.9 → −8.5 μA/cm^2^ at −100 mV, *P* < 0.01), with unchanged L-type Ca^2+^ current, resulting in marked APD_90_ prolongation (0.65 → 1.09 s). These human-derived scaling factors (G_to ≈ 0.60, G_K1 ≈ 0.55–0.60 of control) are frequently adopted in human ventricular cell models (ToR-ORd, Grandi–Bers) to represent chronic post-MI/failing ionic remodeling (54). However, despite being the first study to directly quantify pathological K^+^ current downregulation in isolated human ventricular myocytes, several caveats should be considered when extrapolating these results to post-infarction border zone ionic remodeling in silico. First, the specimens were obtained from the entire left ventricle of patients with end-stage heart failure (NYHA class IV, EF ≈ 25%), neither from acute or subacute infarct border zone (BZ) tissue nor stratified by distance from the infarct core. The measured reductions in Ito and IK1 therefore reflect average chronic global failing or long-standing ICM remodeling, which may not equate to the region-specific ionic alterations occurring days to weeks after MI in the peri-infarct zone. Second, the cohort included both DCM and ICM etiologies, with only six ICM cases that were not further subclassified by infarct location or BZ vs. RZ, potentially masking regional heterogeneity. Third, Cd^2+^ was added and EGTA-based pipette solution strongly buffered intracellular Ca^2+^ to isolate K^+^ currents without interference from Ca^2+^ channels, but this also eliminated Ca^2+^-dependent Ito components and disrupted Ca^2+^-regulated signaling pathways that may influence other currents. Additionally, whole-cell configuration carries an inherent risk of current run-down; although the authors attempted to record within two minutes of membrane rupture, small IK components may still have been underestimated due to rapid decay. Finally, this work focused exclusively on K^+^ currents and action potential morphology, without systematic measurements of late sodium current (INaL), Na^+^/Ca^2+^ exchanger (NCX), or SERCA function—all of which are equally indispensable parameters for a complete description of post-MI electrical remodeling and must be supplemented by other human myocardial studies.

The major post-infarction cellular electrophysiological alterations, their effects on cellular electrophysiology, and the corresponding modeling representations are summarized in Table 3, together with the experimental basis used for their parameterization.

**Table 3:** Cellular electrophysiological remodeling and its representation in post-MI cardiac models.

Cellular parameter/feature	Post-MI/BZ remodeling	Effect on cellular electrophysiology	Modeling representation	Experimental basis
INa	Reduced	Reduced maximum depolarization velocity	Reduced GNa	Patch-clamp measurements
ICaL	Reduced	Altered plateau and Ca^2+^ handling	Reduced GCaL	Patch-clamp measurements
IKr	Moderately reduced	Prolonged repolarization	Reduced GKr	Experimental measurements
IKs	Moderately reduced	Prolonged repolarization	Reduced GKs	Experimental measurements
Ito	Reduced in chronic/failing myocardium	Altered early repolarization	Reduced Gto	Human ventricular myocyte data
IK1	Reduced in chronic/failing myocardium	Altered resting potential and terminal repolarization	Reduced GK1	Human ventricular myocyte data
Calcium handling	Altered	Abnormal Ca^2+^ kinetics/triggered activity	Modified Ca^2+^-handling parameters	Experimental and modeling studies

##### 4.3.2.2 Tissue model parameters and what measurements are available to validate their choices.

At the tissue level, the key model parameters for post-infarction simulations include tissue conductivity, excitability, and the geometric classification of scar and border zone. The electrophysiological impact of fibrosis is commonly represented through modification of tissue conductivity and excitability rather than explicit heterocellular coupling.

Based on the aforementioned modeling approach centered on tissue conductivity and excitability, a widely adopted specific method is the forward simulation using population-averaged conductivities. The most widely adopted framework for simulating electrical wave propagation in infarcted ventricles is the monodomain reaction–diffusion equation, in which intracellular and extracellular conductivities are combined into an effective monodomain conductivity tensor. Orthotropic anisotropy is imposed to reflect myocardial fiber architecture, and infarct scar elements are conventionally treated as electrically non-conductive. In the border zone (grey zone), transverse conductivity is reduced to reflect connexin-43 remodeling, while longitudinal conductivity is relatively preserved. As a representative calibration of this framework, Arevalo et al. (2016) set longitudinal and transverse conductivities in non-infarcted tissue to 0.255 and 0.0775 Sm⁻^1^, respectively, and applied a 90% reduction in transverse conductivity in the grey zone, with no alteration in fiber orientation. These values were fine-tuned to match experimentally measured human ventricular conduction velocity, yielding a longitudinal velocity of approximately 0.6 ms⁻^1^ in normal myocardium. Once electrophysiological parameters are assigned throughout the personalized anatomical mesh, the complete cardiac model is ready for programmed electrical stimulation (52). This forward simulation approach requires no patient-specific electrophysiological data beyond routine LGE−MRI, making it computationally efficient and applicable to large cohorts. It has been validated in multiple studies, demonstrating correct prediction of VT circuit morphology and inducibility in post-infarction patients. However, since the conductivity values are derived from population averages rather than individual measurements, this approach cannot capture inter-patient variability in fibrosis microstructure, gap junction remodeling, or scar composition. Furthermore, the assumption of uniform 90% transverse conductivity reduction across the entire grey zone may oversimplify the spatial heterogeneity of connexin-43 downregulation observed in experimental studies (52).

An alternative parameterization for diffuse microfibrosis was proposed by Alonso et al. (2016). In this approach, fibrotic tissue is represented by randomly removing a fraction ϕ of intercellular conductive links within a specified region, treating fibrotic tissue as a heterogeneous discrete network near the percolation threshold ϕc (Alonso et al., 2016). In this formulation, the connectivity between neighboring myocytes is set to zero with probability ϕ, while normal couplings are preserved with probability 1 − ϕ. Wave propagation is solved on a three-dimensional lattice using a simplified ionic model, and the incidence of microreentry and ectopic pacemaker activity is examined as a function of ϕ and tissue thickness. Results demonstrate that reentrant activity and ectopic foci are most probable when the fraction of non-conducting links is just below the percolation threshold—a finding that informs the interpretation of infarct border zone fibrotic burden in post-myocardial infarction arrhythmogenesis (55). Although Alonso et al. (2016) employed an idealized slab geometry without image-based fibrosis quantification, the underlying principle—representing diffuse microfibrosis as a stochastic removal of intercellular connections with a prescribed fraction near the percolation threshold—can be incorporated into patient-specific infarct border zone models.

A more recent texture-based parameterization was introduced by Lawson et al. (2024). Perlin noise—a procedural noisefield with controllable feature size, anisotropy, and roughness—can be calibrated against histological images of cardiac fibrosis to generate synthetic realisations of physiologically realistic fibrotic patterns, including interstitial, compact, diffuse, and patchy fibrosis subtypes. These patterns are mapped onto the simulation mesh as non-conductive obstacles or used to derive spatially varying conductivity reduction factors via homogenisation, thereby preserving micro-architectural differences (e.g., collagen strands aligned with myofibres vs. scattered micropatches) that influence local conduction velocity and anisotropy. Although such texture-based approaches are not yet standard in patient-specific large-scale simulations, they offer a means to investigate how fine-scale fibrotic architecture contributes to re-entrant substrate formation and may refine the conventional LGE-MRI–derived conductivity reduction strategy in post-infarction models (56).

The effective conductivity tensors used in the Perlin-noise study above were obtained via a volume-averaging homogenisation framework introduced by Lawson et al. (2023) (57). In this standalone method, microscopic non-conductive fibrotic obstacles (∼10 μm resolution) are upscaled to derive an effective conductivity tensor D_eff and a volume fraction ϕ of excitable tissue for each coarse mesh element. This allows sub-mesh-scale effects of collagen deposition—such as conduction slowing, source–sink mismatch, and spiral wave anchoring—to be retained in whole-ventricular simulations with clinically relevant mesh spacings (100–250 μm), and can be applied to infarct border zone fibrosis when micro-architectural patterns are informed by histology or stochastic generators (57).

The four tissue-level fibrosis representation approaches reviewed above are all static in nature. They assign a fixed set of electrical properties to fibrotic and border-zone regions based on a single imaging snapshot or histological classification, and these properties remain unchanged regardless of the activation rate or the coupling interval of premature beats. Despite this simplification, static fibrosis models have achieved notable success: they have been shown to reproduce the induction of monomorphic ventricular tachycardia via programmed electrical stimulation (PES) in patient-specific post-infarction models (52), stratify arrhythmic risk in hypertrophic cardiomyopathy cohorts (58), and predict the exit sites of clinical VT circuits with reasonable accuracy. The majority of clinical validation studies for personalized cardiac simulation in SCD prediction have therefore relied on static fibrosis parameterisations, owing to their computational tractability and compatibility with clinical imaging resolution. Yet, this reliance on static parameterisations should not be mistaken for evidence that the choice among them is inconsequential. The sensitivity of simulation outcome to the static fibrosis schema itself is not trivial. Although derived in the context of atrial fibrillation, Roney et al. (59) demonstrated that applying different static fibrosis representations—percolation, edge splitting, and conductivity scaling—to the same patient LGE-MRI data yielded markedly different phase-singularity counts and rotor anchoring patterns, with only percolation approaching clinically measured values. Notably, mere conductivity reduction combined with ionic remodelling over-localised rotors onto fibrotic patches—a behaviour absent in clinical maps. This methodological sensitivity carries a direct implication for post-MI ventricular modelling: assigning border-zone conductivity by a single static rule, without resolving strand architecture or non-myocyte coupling, may predetermine rotor dynamics irrespective of the true rate-dependent substrate. More fundamentally, a growing body of experimental and computational evidence indicates that static fibrosis representations are fundamentally incapable of reproducing the rate-dependent conduction slowing and block that characterise fibrotic and border-zone tissue.

This limitation originates from the dissociation between structural imaging and the underlying electrophysiological mechanisms. High-resolution mapping of explanted human hearts reveals that the architectural substrate itself dictates rate dependence: premature stimuli at progressively shorter coupling intervals elicit nonlinearly increasing activation delays, a phenomenon tightly linked to the presence of long-strand interstitial fibrosis rather than diffuse collagen deposition (60). Crucially, these delays are strongly anisotropic, scaling with the angle between the wavefront and local myofibre direction. The authors interpreted the anisotropy of the delay as arising from wavefront detours around long collagen strands, path lengthening, and local transitions from longitudinal to transverse propagation—a mechanistic reading that is consistent with their data but was not isolated from possible contributions of diastolic-interval-dependent ionic recovery in the same preparations. Static conductivity-scaling models collapse this architectural specificity into a single isotropic parameter, thereby missing the structural basis of the observed frequency dependence.

The architectural substrate alone, however, does not fully explain the electrophysiological phenotype; it acts as a catalyst that unmasks the intrinsic rate dependence of cardiomyocyte excitability. When diastolic intervals shorten, voltage-gated Na^+^ channels (Nav1.5) have insufficient time to recover from inactivation, and source-sink balance degrades. In a homogeneous sheet, this yields a smooth CV restitution curve, but in a patchy-fibrotic sheet—as characterised by Kawara—the same shortening of coupling interval precipitates abrupt conduction failure at loci where the wavefront crosses long collagen strands transversely. This integrated picture—patchy fibrosis exposing the diastolic-interval dependence of Na^+^-channel recovery—extends beyond what Kawara et al. directly measured, in that their human preparations did not isolate ionic from architectural contributions (the authors explicitly noted that Na^+^-current abnormalities could not be excluded, nor connexin remodelling ruled out). The formulation used here therefore represents a synthesis of their structural finding with independent cellular evidence (e.g., reduced Na^+^ current in infarct border-zone myocytes, as reported by Pu & Boyden (61)), rather than a direct demonstration in their dataset. Accordingly, the experimentally observed rate-dependent conduction slowing likely arises not from collagen *per se*, but from collagen-induced source-sink mismatch that exposes the diastolic-interval dependence of Na^+^-channel recovery. A fibrosis model that holds conductivity constant would be structurally incapable of reproducing this steepening of the activation-delay curve as the coupling interval is decremented.

Compounding this issue, the border zone is not merely a two-component mixture of myocytes and inert collagen; it is a heterocellular microenvironment where non-myocytes actively modulate electrical propagation. Cardiac fibroblasts—and, to a lesser extent, resident macrophages—are documented to form electrotonic couplings with neighbouring myocytes via connexin-43/45 gap junctions *in situ*, whether identical coupling holds in human border zone remains to be established (62). In addition, *in vivo* optogenetic depolarization of scar fibroblasts 10 days to 12 weeks after myocardial infarction elicits organ-wide myocardial excitation and ventricular arrhythmias in mice, an effect that persists after conditional deletion of fibroblast Cx43 (63). These findings collectively establish that non-myocytes can be electrically coupled to myocytes *in vivo* and that this coupling can be functionally consequential. Several aspects of this coupling, however, remain mechanistically unresolved or supported only indirectly. First, ephaptic interactions in narrow perinexal clefts have been proposed as an additional, non-gap-junction route that may operate in tandem with gap junctions, particularly under conditions of compromised coupling; tunnelling nanotubes, although observed morphologically at scar borders by three-dimensional electron tomography, remain a functionally unproven candidate route in scar tissue. Second, because of their non-excitable nature and labile membrane potential, fibroblasts have been modelled and inferred *in vitro* to act as adjustable electrical loads; depending on coupling density and the fibroblast: myocyte ratio—both dynamic after infarction—computational and coculture studies suggest opposing outcomes: sparse coupling partially depolarises attached myocytes and slows conduction, whereas denser fibroblast bridging may relay excitation across collagen gaps (the “repeater station” scenario) or, conversely, dissipate depolarising current and block it (62). Third, when combined with computational modelling, the persistence of optogenetically driven excitation after Cx43 deletion implies that gap-junctional and ephaptic routes can act in a synergistic yet functionally redundant manner to excite coupled myocytes (63)—although the authors note that other routes (e.g., tunnelling nanotubes) or low-abundance connexin-like proteins cannot be fully excluded. Overall, the effective coupling strength is likely shaped by fibroblast membrane potential, connexin expression at heterocellular contacts and local fibroblast density, all of which are dynamic and species-/model-dependent variables that static conductivity maps cannot capture. Treating fibroblasts merely as a conductivity-lowering obstacle therefore discards an actively modulating electrical compartment whose coupling strength is dynamically regulated by membrane potential, connexin availability, and local density—a feature that purely static, time-invariant conductivity models are structurally incapable of representing.

Collectively, these intertwined mechanisms—architectural anisotropy, diastolic-interval-dependent ionic recovery, and heterocellular electrotonic coupling—generate the rate- and premature-beat-dependent conduction behaviour of post-MI border-zone tissue.

A recent methodological advance by Giardini et al. provides a concrete workflow for overcoming the static limitation (64). Using desmoglein-2 mutant mouse hearts—a model of arrhythmogenic cardiomyopathy—they developed a correlative imaging pipeline that integrates whole-ventricle light-sheet microscopy at micrometric resolution with optical mapping of action potential propagation at multiple pacing frequencies. The structural reconstruction distinguishes compact fibrosis (dense collagen patches) from non-compact fibrosis (interstitial/diffuse fibrosis), quantifies three-dimensional cardiomyocyte orientation, and maps connexin-43 distribution within and around fibrotic regions. These structural data are then co-registered with functional activation maps acquired at low frequency (5 Hz) and high frequency (10–15 Hz) pacing, as well as during premature extrasystolic beats.

Crucially, this pipeline revealed that fibrotic tissue behaves as a rate-dependent low-pass filter: conduction proceeds through fibrotic areas at low pacing rates but fails at high rates or at premature coupling intervals shorter than 75–80 ms, creating a functional substrate for reentrant arrhythmias. When the authors fed the high-resolution structural data alone into a monodomain computational model—treating compact fibrosis as non-conductive and non-compact fibrosis as probabilistically non-conducting—the model captured only ∼3–4 ms of the ∼10 ms activation delay observed experimentally at high frequency. Matching the experimental data required the simultaneous incorporation of three electrophysiological alterations: reduced tissue conductivity, reduced sodium channel conductance, and explicit representation of fibroblast–myocyte electrotonic coupling via connexin-43. A one-dimensional myocyte–fibroblast chain model further confirmed that increasing pacing frequency reduces the maximum number of fibroblasts through which excitation can propagate, and decreases both conduction velocity and maximum upstroke velocity. It should be noted, however, that these findings were obtained in a genetic cardiomyopathy model; whether the same rate-dependent filtering behaviour holds in human post-infarction border zone remains to be established.

This study demonstrates that high-resolution structural reconstruction alone, even at the micrometre scale, is insufficient to predict rate-dependent conduction behaviour; the dynamic electrophysiological remodelling of fibrotic tissue—including heterocellular coupling and frequency-dependent ionic recovery—must be explicitly incorporated. For post-infarction border-zone modelling, this implies that future personalised simulations should move beyond static conductivity assignment and adopt a coupled structural–dynamic electrophysiological framework informed by rate-dependent functional readouts. In clinical practice, such models could be personalised using inputs of increasing maturity while remaining compatible with standard workflows: routinely acquired CMR—LGE for scar-core and grey-zone geometry, complemented by native T1/ECV mapping for interstitial fibrosis burden; non-invasive electrical phenotyping from standard 12-lead ECG at rest and during exercise or pharmacological stress, Holter-derived premature-beat dynamics, and signal-averaged ECG where indicated, to constrain rate- and premature-beat-dependent conduction; and higher-density modalities such as ECG imaging or electroanatomical mapping, and tissue-level information (e.g., Cx43 or fibroblast activation markers) obtained from endomyocardial biopsy where clinically feasible, serving as calibration or validation layers rather than routine inputs. Together, these data would allow the model to be personalised—without requiring *ex vivo* optical mapping—while retaining compatibility with clinical routines, thereby improving fidelity to the dynamic arrhythmic substrate that governs arrhythmia initiation under exercise or premature beats.

The major tissue-level electrophysiological parameters, their computational representations, and the principal strategies used to model post-infarction tissue are summarized in Table 4.

**Table 4:** Tissue-level electrophysiological parameters and modeling strategies in post-MI ventricles.

Tissue-level component	Modeling representation	Typical approach described in the manuscript	What it captures	Main limitation
Tissue conductivity	Anisotropic conductivity tensor	Monodomain reaction–diffusion model	Direction-dependent electrical propagation	Often based on population-averaged values
Scar conductivity	Markedly reduced or non-conductive	Scar assigned low/non-conductive properties	Electrical isolation of dense scar	May oversimplify residual conduction
Border-zone conductivity	Reduced regional conductivity	Reduced transverse conductivity with relatively preserved longitudinal conductivity	Conduction slowing and anisotropy	May not capture spatial heterogeneity
Fibrosis/microfibrosis	Discontinuous conductive network	Stochastic removal of intercellular conductive links	Microstructural connectivity	Often based on idealized tissue assumptions
Fibrosis texture	Spatially heterogeneous tissue pattern	Texture-based representation	Heterogeneous fibrosis architecture	More computationally demanding
Effective tissue properties	Homogenized conductivity	Homogenization approach	Sub-mesh microstructural effects	Depends on microstructural assumptions
Excitability	Cellular/tissue electrophysiological properties	Regional modification of electrophysiological parameters	Regional differences in propagation	Direct patient-specific measurements are limited

##### 4.3.2.3 Parameter estimation techniques that have been explored for cell or tissue model parameters.

Beyond directly adopting experimentally measured or extrapolated conductance scaling factors, a complementary family of techniques estimates cell or tissue model parameters by fitting model outputs to observed data.

Beyond the classical forward simulation approach, an increasingly adopted strategy for personalising the conduction velocity field is to calibrate tissue conductivity parameters directly from intraprocedurally acquired electroanatomical mapping data. In this inverse-type workflow, the anisotropic conductivity tensor—or equivalently the local conduction velocity tensor—is adjusted so that the simulated activation times, typically obtained from an eikonal or monodomain forward solve, minimise the least-squares mismatch with clinically recorded local activation times across the mapped cardiac surface. Iterative tuning methods, such as those implemented in pipelines like AugmentA, locally modify element-wise conductivities to reduce the root-mean-square error between simulated and measured activation times, while conduction-velocity-based calibration derives bulk orthotropic conductivities by matching one-dimensional cable-derived CVs to those estimated from EAM isochrone maps. More recent inverse eikonal techniques, such as the Personalized Inverse Eikonal Model from Cardiac Electro-Anatomical Maps, formulate the problem as a regularised inverse optimisation in which the conductivity tensor field is recovered globally, with fibre direction and CV magnitude extracted via eigen-decomposition of the tensor. The principal advantage of EAM-based calibration lies in its ability to yield patient-specific conduction properties without relying exclusively on population-averaged conductivity values, thereby accounting for inter-individual heterogeneity in fibrosis and microstructural remodelling. Additionally, eikonal-based inverse solvers are computationally efficient and can operate on sparse or noisy mapping data, making them feasible for integration into clinical workflows. Nevertheless, several limitations warrant consideration. First, the quality of the calibration outcome is heavily dependent on the spatial coverage and density of the clinical EAM dataset; in regions where mapping points are sparse, the reconstructed conductivity field may be poorly constrained, leading to non-unique or physiologically implausible solutions. Second, the recovered conduction velocity values are inherently tied to the specific pacing protocol and wavefront direction used during mapping, and whether these calibrated parameters generalise to arrhythmic scenarios involving different activation patterns or reentrant circuits remains an open question. Third, the computational cost of iterative tuning methods, particularly when applied to high-resolution volumetric meshes with repeated calibrations for multiple pacing sites, can become prohibitive, limiting their scalability for large-cohort studies. Current applications of EAM-calibrated models include the reconstruction of patient-specific activation patterns for atrial fibrillation substrate characterisation, guidance of ablation lesion placement through in silico scenario testing, and the generation of cardiac digital twins for arrhythmia risk stratification (65).

Beyond patient-specific EAM calibration， the effective conductivity tensors used as input to this framework can also be derived from alternative sources, such as volume-averaged homogenisation of micro-scale fibrotic structures (57). These techniques, however, do not replace the classical monodomain-based calculation of conduction velocity described above.

Recent studies have explored complementary inverse-engineering strategies for post-MI electrophysiology that do not themselves supply independent ionic remodeling parameter sets. Others use deep-learning surrogate models to infer infarct anatomy and regional conduction velocity reduction (scar CV × 0.1, border-zone CV × 0.5 of normal) from 12-lead ECG, bypassing ionic-level parameter estimation altogether (66). Although these approaches accelerate the cardiac inverse problem and enable patient-specific anatomical or tissue-level characterization, they currently stop short of resolving cell-level ionic conductance scaling—such as border-zone G_Na, G_Kr or G_to downregulation—and thus remain computational refinements of, rather than alternatives to, experimentally anchored ionic remodeling schemes.

At the cellular level, parameter estimation techniques have also been developed to determine ionic conductance scaling factors from experimental or clinical data. A widely adopted strategy is to treat maximal conductances as unknown parameters and calibrate them against experimentally or clinically observed cellular or tissue-level phenotypes. Whittaker et al. reviewed several approaches along this line. Global optimization methods, such as genetic algorithms and Markov chain Monte Carlo, iteratively adjust conductance values until the simulated action potential duration, upstroke velocity, calcium transient and drug-block responses collectively match corresponding experimental measurements. The Population of Models approach samples conductances over physiologically plausible ranges, generates thousands of virtual cells, and retains only those parameter sets whose simulated outputs fall within published normal or border zone biomarker intervals, thereby defining a feasible parameter space that accommodates inter-subject variability. Recalibration adjusts an existing model's conductances to fit a new disease condition or patient-specific dataset by re-running the optimization against target phenotypes (67). Despite their utility, these methods share important limitations: the inverse problem is inherently ill-posed, meaning multiple distinct combinations of conductance changes can produce nearly identical action potentials; the resulting admissible ranges are sensitive to the choice and quality of the validation biomarkers; most implementations assume independent variation of each conductance unless co-regulation constraints are explicitly introduced; and the derived ranges represent feasible solutions rather than direct molecular evidence of channel remodeling.

Zhou et al. directly embedded the POM framework within a multi-source experimental and clinical validation pipeline specifically designed for post-infarction ionic remodeling. The Population of Models (POM) framework samples ionic current conductances within physiological ranges and calibrates the resulting parameter sets against experimental biomarkers, thereby capturing inter-subject variability in tissue- and organ-level simulations. This approach synthesizes heterogeneous experimental data—including patch-clamp recordings from canine and minipig infarct border zones, ion channel protein expression assays from failing human myocardium, and action potential clamp-derived current decompositions—to define multiple gradations of ionic remodeling corresponding to acute and chronic post-MI stages (68). Specifically, acute infarct border zone (BZ) remodeling is represented by three variant schemes (Acute BZ1–BZ3) reflecting inter-patient variability: all schemes impose a marked reduction in the fast sodium current (*G*_Na_ scaled to 0.38–0.40 of control) and L-type calcium current (*G*_CaL_ to 0.31–0.64), with differential downregulation of rapid delayed rectifier potassium current (*G*_Kr_ to 0.30–0.70) and near-abolition of transient outward potassium current (*G*_to_ to 0–0.10). Chronic remodeling incorporates partial recovery of *G*_Kr_ and *G*_Ks_ in the BZ, alongside late sodium current upregulation (*G*_NaL_ × 1.275– × 2.0) and SERCA pump suppression (*J*_up_ × 0.4), while remote zone (RZ) remodeling in the chronic phase applies additional *I*_Kr_ inhibition (to 0.60–0.87) to simulate global repolarization prolongation. These parameterized schemes are further validated by constraining simulated action potential duration (APD_90_), calcium transient amplitude, and 12-lead ECG phenotypes (QT interval, T-wave morphology) against experimental and clinical post-MI data. The use of multiple BZ/RZ configurations captures the electrophysiological heterogeneity and phenotypic variability observed across patients, thereby providing a physiologically grounded basis for parameter selection beyond single-species patch-clamp extrapolation.

The main approaches explored for estimating and personalizing cellular and tissue electrophysiological parameters, together with their data requirements and limitations, are summarized in Table 5.

**Table 5:** Parameter estimation and personalization approaches for cardiac electrophysiological modeling.

Approach	Input data	Parameters estimated/calibrated	Basic principle	Main advantage	Key limitation
Population-based parameterization	Literature/experimental data + patient-specific anatomy	Cellular and tissue electrophysiological parameters	Assign population-derived values to the personalized anatomical model	Simple and computationally efficient	Limited patient specificity
Experimental scaling	Patch-clamp/experimental measurements	Ion-channel conductances	Apply experimentally derived scaling factors to baseline cell models	Direct physiological basis	Human post-MI BZ measurements are limited
Phenotype-based calibration	AP morphology, APD, Ca^2+^ handling or other electrophysiological phenotypes	Cellular ionic parameters	Adjust parameters until simulated phenotypes match observations	Can reproduce measured electrophysiological behavior	Inverse problem may be ill-posed
EAM-based inverse calibration	Electroanatomical mapping/activation times	Tissue conductivity or conduction velocity	Optimize conductivity to minimize simulated–measured activation-time mismatch	Patient-specific conduction properties	Dependent on mapping coverage and quality
Inverse eikonal/conductivity reconstruction	EAM activation maps + anatomical model	Conductivity tensor/CV field	Regularized inverse optimization	Computationally efficient for conduction calibration	Solutions may be non-unique
AI-assisted inference	ECG/multimodal clinical data	Anatomical or regional electrophysiological features	Learn relationships between clinical data and model parameters/features	Rapid inference and multimodal integration	Requires extensive validation and interpretability remains limited

### 4.4 Prediction

The central predictive paradigm of personalized cardiac simulation modeling is the in silico programmed electrical stimulation (PES) protocol. The electrophysiological structure of the model, manifested as the electrical activity at the organ level, requires computer induction to simulate the patient's cardiac physiological state. The model is subsequently validated by comparing its outputs with the patient's actual clinical data. To maximally replicate the clinical process of inducing malignant ventricular arrhythmias in a catheterization laboratory setting, programmed electrical stimulation (PES) can be applied at 19 pre-defined anatomical sites. These sites are strictly defined according to the American Heart Association (AHA) standards, with 17 located in the left ventricle, one in the right ventricular outflow tract, and one at the right ventricular apex. This protocol mimics the conditions of premature beats, aiming to reveal potential reentrant circuits or triggered activity arising from the personalized substrate of myocardial fibrosis and electrophysiological heterogeneity. The programmed electrical stimulation protocol consists of two phases: a baseline pacing train, followed by extrastimuli. The baseline train, typically delivered at the right ventricular apex, involves a series of stimuli to bring the heart model to an electrophysiological steady state, eliminating memory effects and standardizing action potential morphology. Subsequently, a series of extrastimuli with progressively shorter coupling intervals are introduced until the refractory period is encountered or a reentrant arrhythmia is induced (69). Comparing the simulation results, such as the inducibility of arrhythmias, with the patient's actual clinical data (e.g., results from an invasive electrophysiology study or documented spontaneous arrhythmias) allows for the assessment of the model's accuracy, sensitivity, and specificity. This comparative validation enables iterative refinement of the electrophysiological parameters, narrowing down the parameter adjustment range to achieve a model of higher fidelity. If the stimulation protocol induces malignant ventricular arrhythmias like ventricular tachycardia or fibrillation in the simulation, the patient's computational heart model is flagged as “susceptible,” thereby classifying the individual as high-risk for sudden cardiac death. This strategy significantly enhances the sensitivity and reliability of the virtual assay, providing a robust technical foundation for non-invasive risk stratification.

While in silico PES provides the mechanistic gold standard for SCD risk prediction by simulating dynamic arrhythmogenesis, image-derived metrics such as GDS and LGE–myocardium interface area, together with computational substrate analyses such as VITA re-entrant pathway detection, offer complementary, continuous prognostic descriptors that capture distinct aspects of fibrotic substrate complexity across ischemic and non-ischemic cardiomyopathies.

The Global Heterogeneity Score (GDS) is an emerging quantitative analysis tool based on LGE cardiac magnetic resonance imaging. Its core principle involves predicting arrhythmic risk by assessing the spatial heterogeneity and complexity of signal intensity within the fibrotic (scar) region. The specific operational procedure utilizes post-processing software to perform a pixel-level analysis of the signal intensity variation between each enhanced voxel in the LGE image and its surrounding tissue. This analysis calculates a quantitative score that reflects the degree of structural disorganization throughout the scar region. The result is an objective, continuous numerical index. A higher GDS indicates a more unstable electrical conduction environment within the fibrotic tissue, which is more prone to forming reentrant circuits. Crucially, the GDS provides incremental prognostic information independent of the traditional measure of fibrosis “burden” (such as the percentage of left ventricular mass). This allows for more precise identification of patients who, despite appearing low-risk by conventional assessments, may harbor a high risk of sudden death. If the resulting burden percentage exceeds a predefined risk threshold, it holds significant clinical implications for SCD risk stratification (42).

The VITA framework begins by reconstructing patient-specific ventricular models from LGE images, from which three-dimensional surface metrics—particularly the interface area between healthy myocardium and total scar, and between the border zone and scar core—are quantified. A fast eikonal-based propagation model is then applied to simulate wavefront propagation through the personalized substrate. By sequentially initiating paced wavefronts from multiple anatomical sites, the algorithm automatically detects electrically isolated isthmuses within the infarct region and computes the corresponding reentrant pathways. For each identified circuit, the round trip time (RTT), representing the computational analogue of the tachycardia cycle length, is calculated. Key output metrics include the total number of in silico inducible ventricular tachycardias, the number of unique VTs, and the mean and maximum RTT. These metrics have been demonstrated to be independently associated with appropriate ICD therapy in post-infarction patients, providing continuous, mechanism-informed substrate descriptors that augment the prognostic value of conventional LGE volumetry and binary PES inducibility (70).

For non-ischemic dilated cardiomyopathy (NIDCM) in which classical infarct border-zone definitions may be ambiguous, the three-dimensional surface area of the interface between late gadolinium enhancement (LGE)–defined fibrotic regions and adjacent viable myocardium can be quantified directly from the segmented anatomical model. This interface metric captures the abrupt transition in tissue properties conducive to rate-dependent unidirectional conduction block and micro-reentry. To complement image-based descriptors, simplified monodomain or bidomain simulations—typically applied to short-axis LGE-derived 2-D triangular meshes with scar conductivities modulated to reflect patchy fibrosis—may be used to apply localized programmed electrical stimulation and enumerate the number of successfully induced re-entrant activations per patient. Both the LGE–myocardium interface area and the count of simulated re-entries have been shown to be independently associated with appropriate ICD therapy and major arrhythmic events in NIDCM cohorts, providing substrate-specific prognostic information that augments conventional LGE volume and LVEF-based risk assessment (71).

The overall workflow of personalized cardiac computational modeling is summarized in Figure 3. By integrating patient-specific imaging, electrophysiological, and clinical information, these approaches enable the construction of individualized cardiac models for in silico assessment of electrical propagation and arrhythmia susceptibility. Virtual electrophysiological testing can subsequently provide mechanistic and quantitative information regarding arrhythmogenic substrates and reentrant activity, which may complement conventional risk markers and contribute to more personalized SCD risk stratification and clinical decision-making.

**Figure 3:**
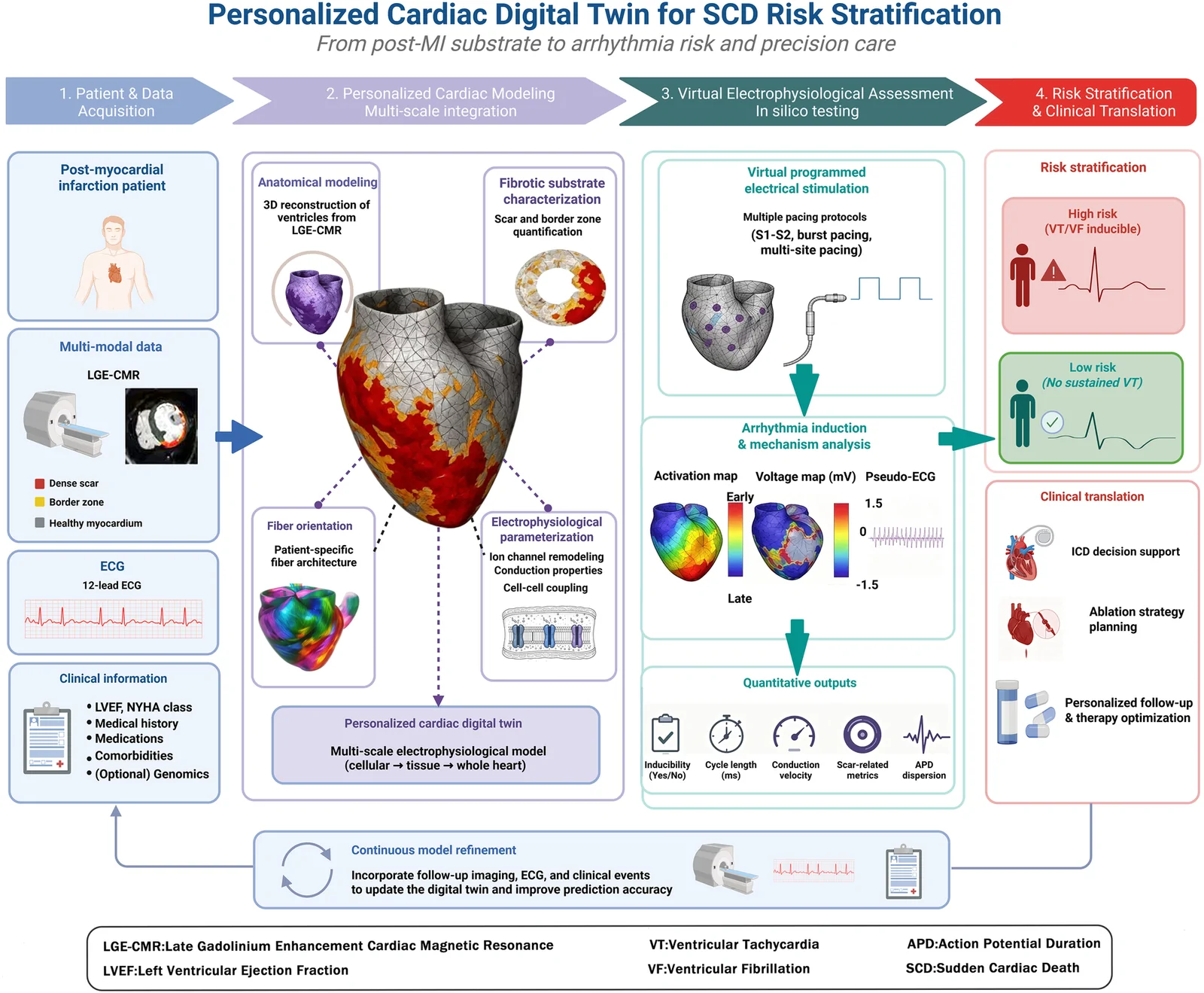
Overview of personalized cardiac computational modeling for sudden cardiac death risk stratification: patient-specific information, including late gadolinium enhancement cardiac magnetic resonance (LGE-CMR), electrocardiographic data, and relevant clinical characteristics, provides the basis for model construction. LGE-CMR data are used to reconstruct patient-specific ventricular anatomy and characterize the fibrotic substrate, including scar and border-zone regions, which can subsequently be incorporated into a three-dimensional computational mesh with myocardial fiber orientation. Electrophysiological parameters are then assigned to the anatomical model using appropriate cellular and tissue-level representations to account for post-infarction electrophysiological heterogeneity. The resulting personalized cardiac model enables in silico electrophysiological assessment through virtual programmed electrical stimulation and simulation of electrical wave propagation, facilitating the identification of arrhythmia inducibility, reentrant pathways, and other quantitative descriptors of the arrhythmogenic substrate. These simulation-derived findings can complement conventional clinical and imaging-based assessment to support individualized SCD risk stratification and clinical decision-making.

### 4.5 Applications

Cardiac simulation modeling has been applied across multiple domains of arrhythmia research. We do not aim to provide an exhaustive listing—which would be impractical given the breadth of the field—this section highlights representative studies that illustrate three key application tiers: model construction and validation standards, mechanistic investigation of arrhythmogenesis, and clinical translation for risk stratification and treatment guidance.

In the domain of model construction and validation, continuous refinements are being made to the model construction process itself. For instance, the Department of Mechanical Engineering at the University of California has established clinical validation standards for assessing model accuracy (72), while researchers like Elham Behradfar et al. have investigated the role of Purkinje-myocardial coupling in ventricular arrhythmias and attempted corresponding modeling (73). From a mechanistic perspective, Mark A. Walker et al. utilized calcium release models to study the ionic mechanisms underlying delayed afterdepolarizations (DADs) (74), and Hermenegild Arevalo et al. examined the sustenance of reentrant circuits in infarcted hearts (75). On the clinical translation front, Johns Hopkins University explored the feasibility of using image-based modeling for assessing ablation targets in ventricular arrhythmias (76), and researchers like Yuanfang X. and Eleonora Grandi et al. studied the impact of β-adrenergic receptor agonists on the transition from ventricular tachycardia to fibrillation (77).

A unified gold standard for the clinical prediction of ventricular arrhythmias using cardiac simulation modeling remains to be established, and this approach has not yet been incorporated into routine clinical practice (78). However, retrospective and prospective studies conducted across multiple centers have demonstrated its clinical validity. In a study with appropriate ICD therapy and cardiac death as primary endpoints, a positive result in the simulation model test was significantly associated with the outcomes and outperformed clinical metrics including LVEF, gray zone volume, scar volume, and left ventricular mass (52); furthermore, research has focused on predicting the risk of recurrence after ablation in patients with post-infarction ventricular tachycardia (VT). The results indicated that the predictive capability of the VITA metric—a computational tool for automatically identifying potential reentrant pathways—demonstrated a stronger association with the endpoint (post-ablation sustained VT requiring ICD therapy or hospitalization) compared to traditional LGE−CMR imaging metrics such as border zone mass or channel mass. This suggests that LGE-MRI-based simulation modeling via the VITA computational framework is able to effectively assess the complexity of the VT substrate and accurately predict the risk of recurrence following catheter ablation (79). For the prediction of non-infarct-related ventricular arrhythmias, research in patients with hypertrophic cardiomyopathy (HCM) has demonstrated that a method constructing personalized computational heart models by integrating LGE−CMR and T1 mapping data successfully achieves precise prediction of ventricular arrhythmia (VA) risk, with performance significantly outperforming existing guideline-based approaches (58). These studies provide a powerful new tool for optimizing the precision of implantable cardioverter-defibrillator (ICD) placement and avoiding unnecessary therapy.

## 5 Challenges and future perspectives

Despite increasing evidence supporting the potential of personalized cardiac computational modeling, several challenges continue to limit its translation into routine clinical practice. These challenges also define the major directions for future development, which is shown in Figure 4. Simulation modeling technology faces several challenges requiring resolution. Firstly, multi-scale modeling is highly complex. The parameters used at different modeling scales span orders of magnitude, and the quantitative relationships between microscopic parameters and macroscopic phenomena are not fully established. High-resolution whole-heart models contain millions of grid nodes, and solving the complex system comprising dozens of differential equations demands high-performance computing, posing significant hardware and software requirements. Temporally, electrophysiological processes are modeled in milliseconds, while tissue remodeling occurs over days, weeks, or even months; the coupling algorithms for these disparate time scales remain in their early stages. Secondly, ensuring accuracy in personalized modeling needs improvement. For instance, the semi-automatic segmentation of MRI data for accuracy still relies on manual point selection, introducing potential bias. During electrophysiological parameter calibration, the process depends on ion channel conductance ranges defined by laboratory patch-clamp techniques rather than direct individual measurements, limiting its suitability for precise personalized modeling. When validating endpoint events, the lack of a universally accepted gold-standard biomarker for sudden cardiac death may lead to a discrepancy between the timing set for endpoint events and the actual timing of structural or functional changes.

**Figure 4:**
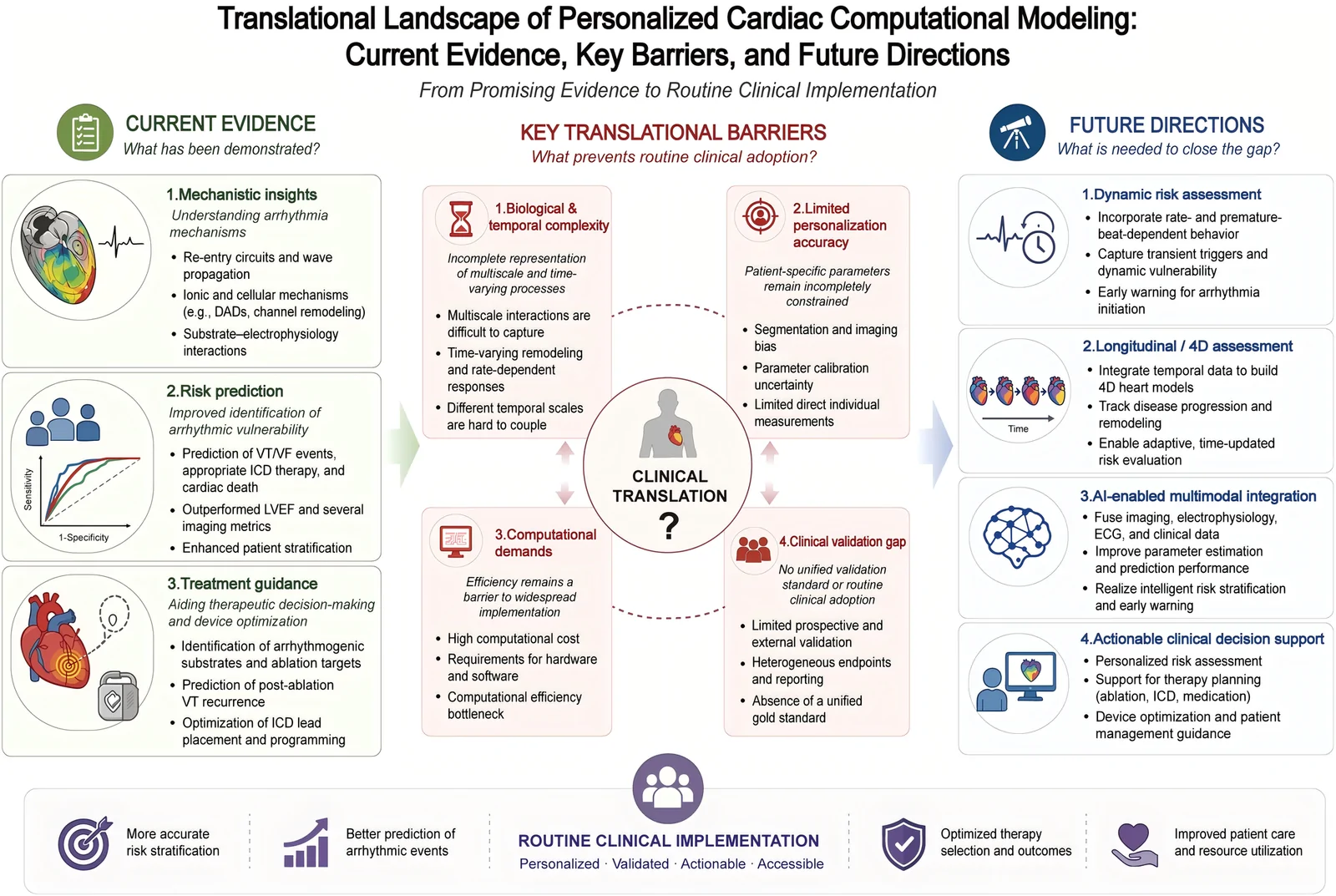
Translational landscape of personalized cardiac computational modeling: current evidence, key barriers, and future directions for translating personalized cardiac computational modeling from research applications toward routine clinical implementation.

Beyond these technical challenges, the clinical need for more precise characterization of the arrhythmogenic substrate is also increasingly recognized.

Recent evidence from the CMR GUIDE randomized clinical trial further highlights the potential clinical relevance of myocardial scar beyond LVEF-based risk stratification. In this trial, 353 patients with mild-to-moderate LV systolic dysfunction (LVEF 36%–50%) and CMR-detected myocardial scar were randomized to receive a primary-prevention ICD or an implantable loop recorder. The primary composite outcome of sudden cardiac death or hemodynamically significant ventricular arrhythmia was not significantly reduced by ICD implantation (7.8% vs. 9.2%; HR 0.76, 95% CI 0.37–1.58). However, sudden cardiac death as an individual outcome occurred less frequently in the ICD group (1.7% vs. 5.8%; HR 0.26, 95% CI 0.07–0.95). These findings provide contemporary randomized evidence that myocardial scar identified by CMR may contain clinically relevant arrhythmic information in patients with LVEF above the conventional ICD threshold, while also indicating that scar-based selection alone may not fully capture the heterogeneous electrophysiological substrate underlying ventricular arrhythmias (80).

This limitation is particularly relevant to personalized cardiac modeling, which can extend beyond structural scar characterization by integrating patient-specific anatomy with tissue conduction properties, cellular electrophysiology, and dynamic arrhythmogenic mechanisms. Such an approach may provide a more comprehensive representation of the substrate responsible for ventricular arrhythmia and may complement imaging-based risk stratification in future clinical applications.

Based on existing research, future studies on ventricular arrhythmia prediction will trend towards multi-dimensional integration. In model construction, there is a need to develop more refined multi-scale electrophysiological-mechanical coupling models that incorporate the spatial distribution of fibrotic foci and the resulting anisotropic conduction. Regarding AI integration, efforts should focus on developing deep learning-based dynamic risk assessment systems that fuse multi-modal data to establish early warning models for ventricular arrhythmias. Key technical bottlenecks to address include automated CMR image segmentation and computational efficiency, alongside integrating temporal data to create cardiac 4D models. Clinical translation will focus on three main directions: personalized risk assessment, aiming for cross-scale prediction from genetic mutations (e.g., SCN5A, KCNH2) to abnormalities in tissue-level electrical activity; treatment decision support systems capable of simulating the blocking effect of ablation lines on reentrant circuits (conduction block zone >5 mm) and the targeted effects of antiarrhythmic drugs (e.g., the inhibition rate of late sodium current by I_Na blockers); and device optimization platforms to quantify the adaptability of ICD therapy strategies to different substrate characteristics (scar burden, border zone geometry). These advancements require interdisciplinary collaboration, including developing smart biomaterials with myocardium-mimetic properties (tunable Young's modulus 10–100 kPa), high-density microelectrode arrays (spatial resolution <100 µm), and novel parallel computing architectures (GPU/quantum computing acceleration), ultimately achieving a paradigm shift from “passive treatment” to “active prevention.”
